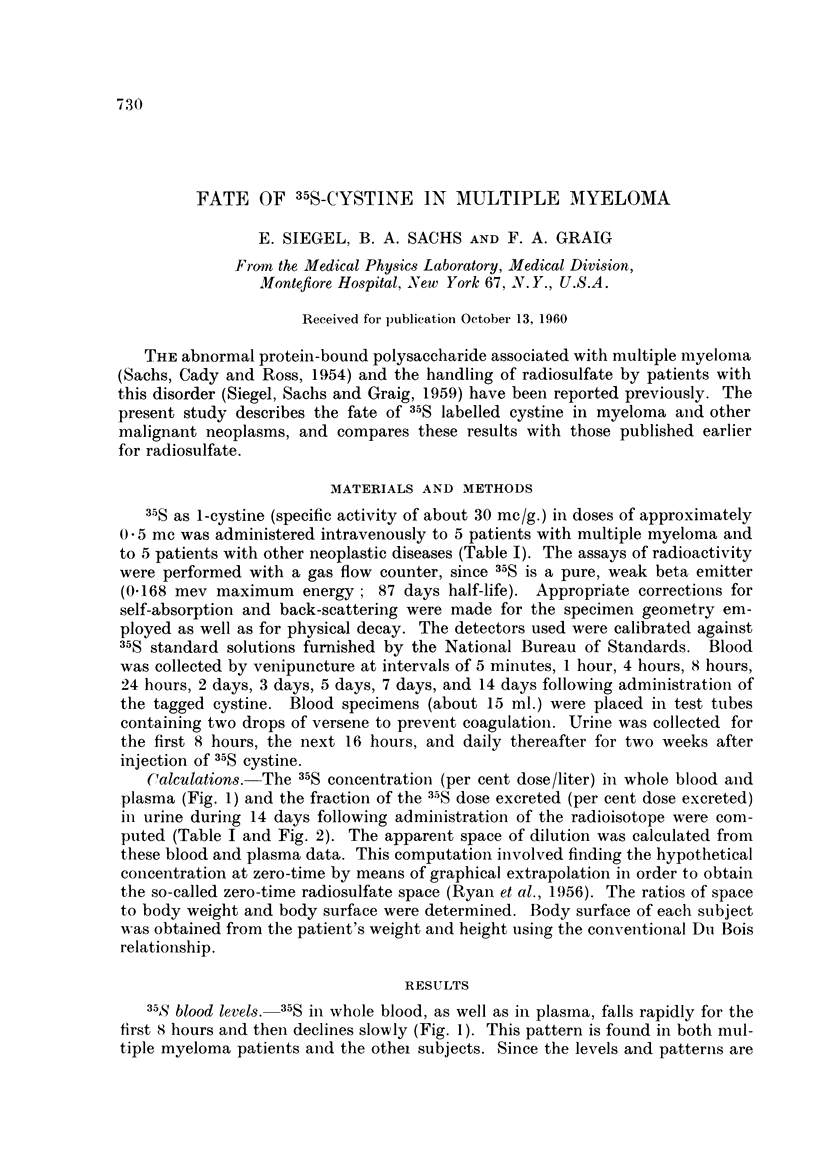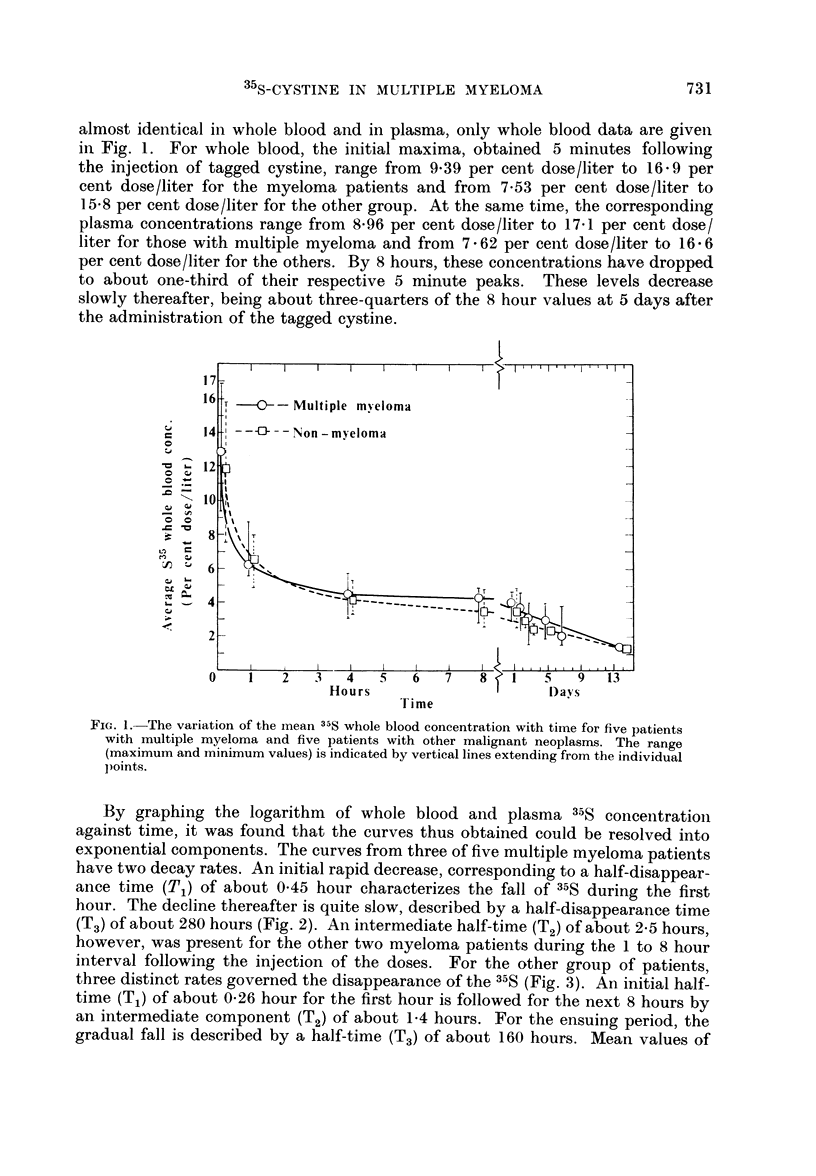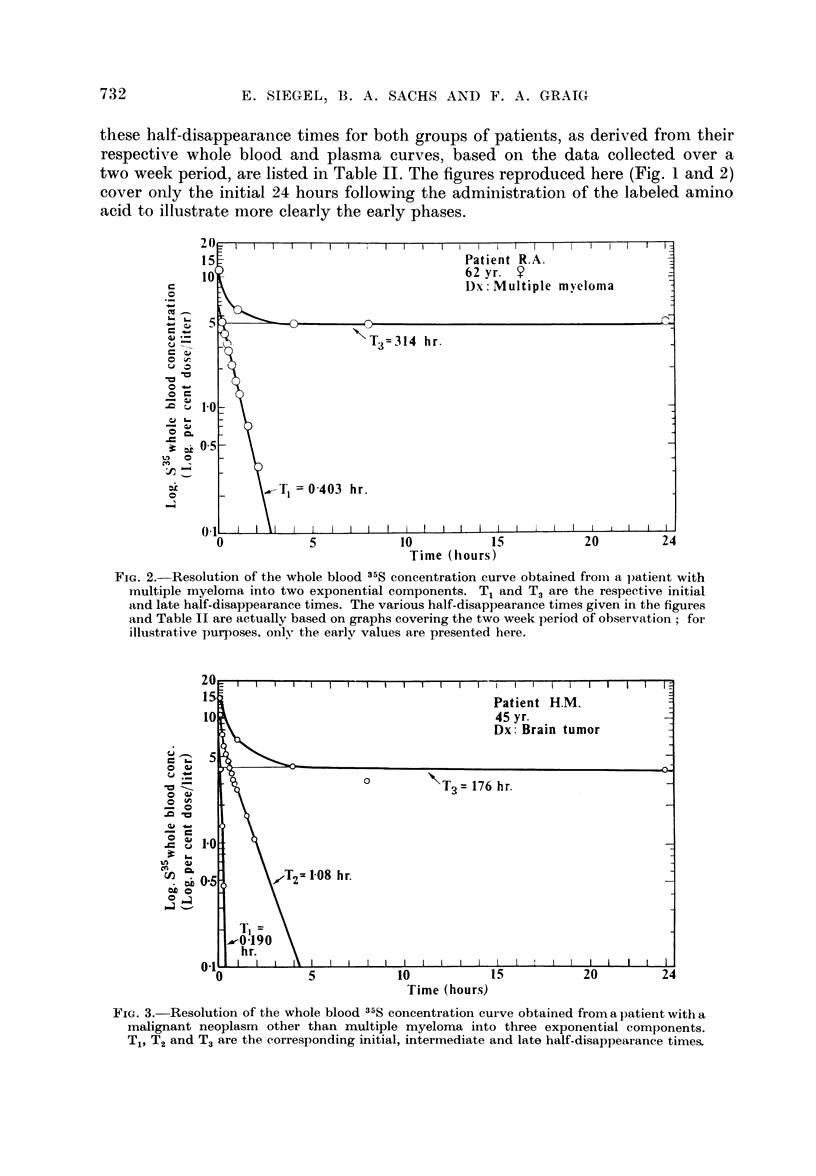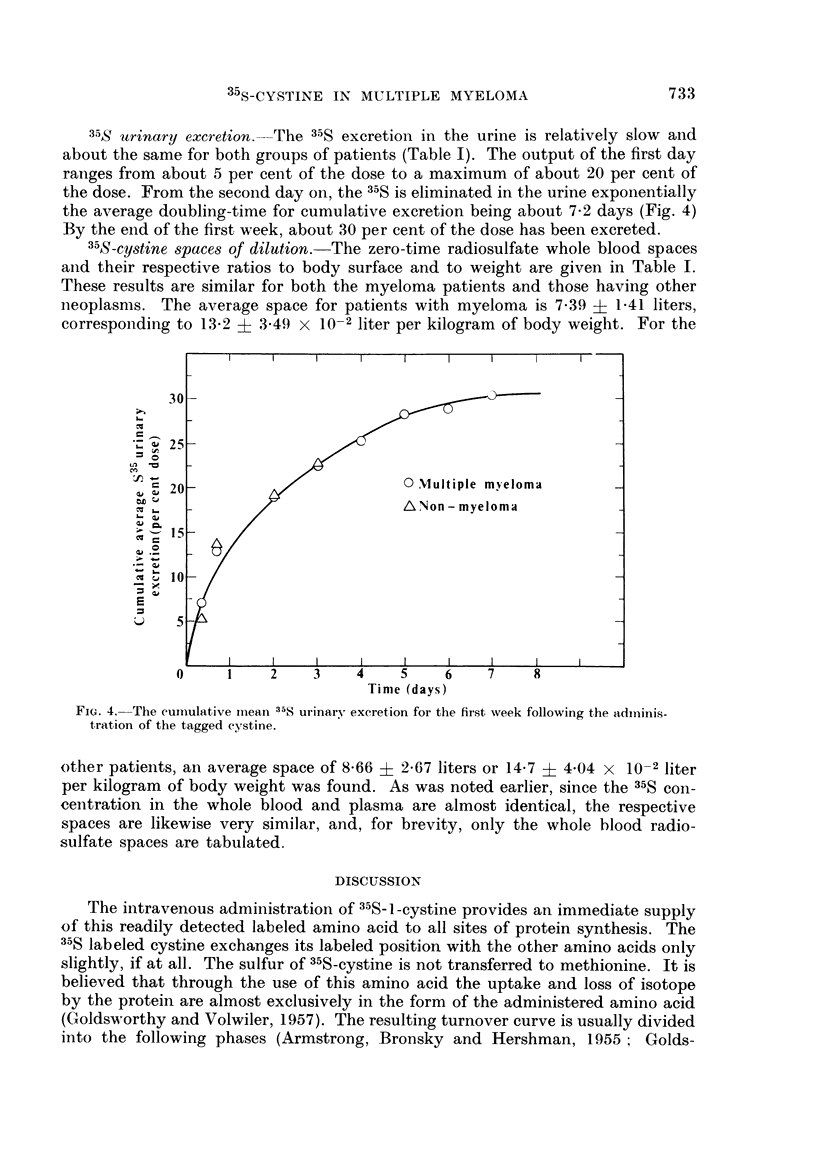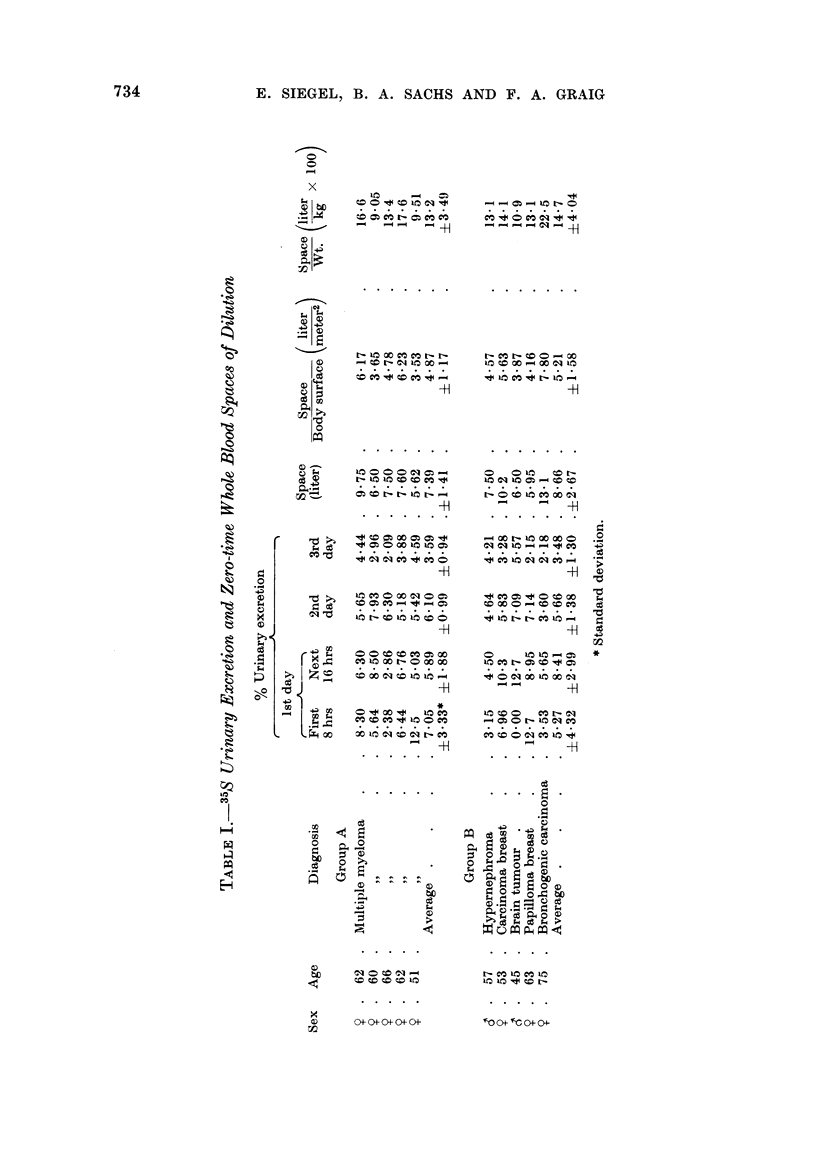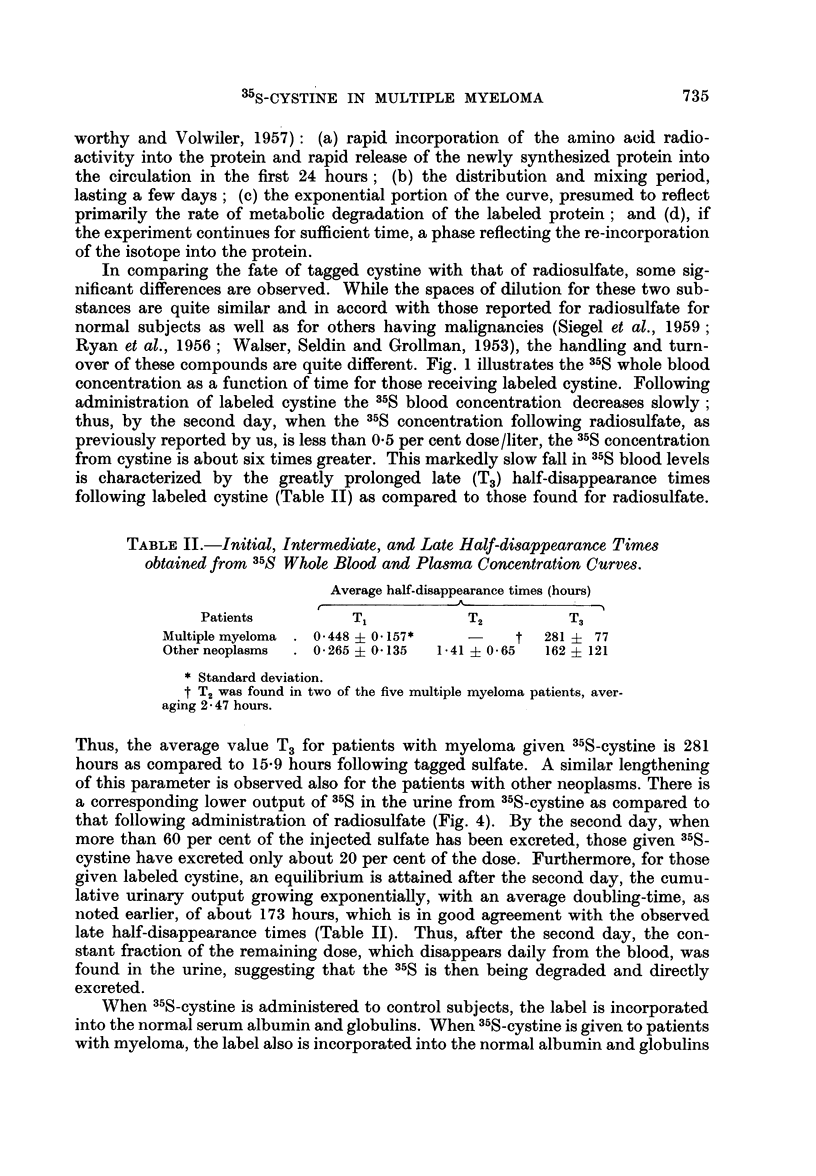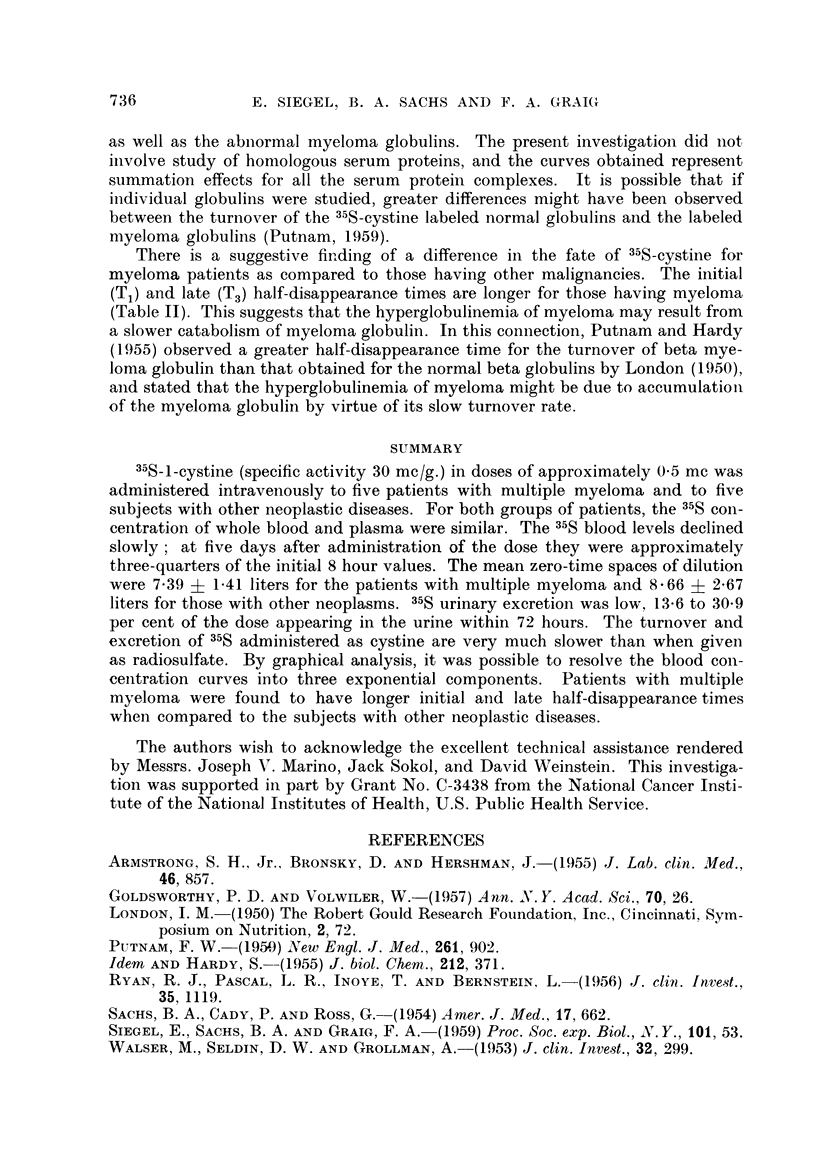# Fate of ^35^S-Cystine in Multiple Myeloma

**DOI:** 10.1038/bjc.1960.83

**Published:** 1960-12

**Authors:** E. Siegel, B. A. Sachs, F. A. Graig


### FATE OF $^{35}$S-CYSTINE IN MULTIPLE MYELOMA

E. SIEGEL, B. A. SACHS AND F. A. GRAIG

*From the Medical Physics Laboratory, Medical Division, Montefiore Hospital, New York 67, N.Y., U.S.A.*

Received for publication October 13, 1960

THE abnormal protein-bound polysaccharide associated with multiple myeloma (Sachs, Cady and Ross, 1954) and the handling of radiosulfate by patients with this disorder (Siegel, Sachs and Graig, 1959) have been reported previously. The present study describes the fate of $^{35}$S labelled cystine in myeloma and other malignant neoplasms, and compares these results with those published earlier for radiosulfate.

### MATERIALS AND METHODS

$^{35}$S as l-cystine (specific activity of about 30 mc/g.) in doses of approximately 0·5 mc was administered intravenously to 5 patients with multiple myeloma and to 5 patients with other neoplastic diseases (Table I). The assays of radioactivity were performed with a gas flow counter, since $^{35}$S is a pure, weak beta emitter (0·168 mev maximum energy ; 87 days half-life). Appropriate corrections for self-absorption and back-scattering were made for the specimen geometry employed as well as for physical decay. The detectors used were calibrated against $^{35}$S standard solutions furnished by the National Bureau of Standards. Blood was collected by venipuncture at intervals of 5 minutes, 1 hour, 4 hours, 8 hours, 24 hours, 2 days, 3 days, 5 days, 7 days, and 14 days following administration of the tagged cystine. Blood specimens (about 15 ml.) were placed in test tubes containing two drops of versene to prevent coagulation. Urine was collected for the first 8 hours, the next 16 hours, and daily thereafter for two weeks after injection of $^{35}$S cystine.

*Calculations.*—The $^{35}$S concentration (per cent dose/liter) in whole blood and plasma (Fig. 1) and the fraction of the $^{35}$S dose excreted (per cent dose excreted) in urine during 14 days following administration of the radioisotope were computed (Table I and Fig. 2). The apparent space of dilution was calculated from these blood and plasma data. This computation involved finding the hypothetical concentration at zero-time by means of graphical extrapolation in order to obtain the so-called zero-time radiosulfate space (Ryan *et al.*, 1956). The ratios of space to body weight and body surface were determined. Body surface of each subject was obtained from the patient's weight and height using the conventional Du Bois relationship.

### RESULTS

*$^{35}$S blood levels.*—$^{35}$S in whole blood, as well as in plasma, falls rapidly for the first 8 hours and then declines slowly (Fig. 1). This pattern is found in both multiple myeloma patients and the othei subjects. Since the levels and patterns are almost identical in whole blood and in plasma, only whole blood data are given in Fig. 1. For whole blood, the initial maxima, obtained 5 minutes following the injection of tagged cystine, range from 9·39 per cent dose/liter to 16·9 per cent dose/liter for the myeloma patients and from 7·53 per cent dose/liter to 15·8 per cent dose/liter for the other group. At the same time, the corresponding plasma concentrations range from 8·96 per cent dose/liter to 17·1 per cent dose/liter for those with multiple myeloma and from 7·62 per cent dose/liter to 16·6 per cent dose/liter for the others. By 8 hours, these concentrations have dropped to about one-third of their respective 5 minute peaks. These levels decrease slowly thereafter, being about three-quarters of the 8 hour values at 5 days after the administration of the tagged cystine.

FIG. 1.—The variation of the mean $^{35}$S whole blood concentration with time for five patients with multiple myeloma and five patients with other malignant neoplasms. The range (maximum and minimum values) is indicated by vertical lines extending from the individual points.

By graphing the logarithm of whole blood and plasma $^{35}$S concentration against time, it was found that the curves thus obtained could be resolved into exponential components. The curves from three of five multiple myeloma patients have two decay rates. An initial rapid decrease, corresponding to a half-disappearance time ($T_1$) of about 0·45 hour characterizes the fall of $^{35}$S during the first hour. The decline thereafter is quite slow, described by a half-disappearance time ($T_3$) of about 280 hours (Fig. 2). An intermediate half-time ($T_2$) of about 2·5 hours, however, was present for the other two myeloma patients during the 1 to 8 hour interval following the injection of the doses. For the other group of patients, three distinct rates governed the disappearance of the $^{35}$S (Fig. 3). An initial half-time ($T_1$) of about 0·26 hour for the first hour is followed for the next 8 hours by an intermediate component ($T_2$) of about 1·4 hours. For the ensuing period, the gradual fall is described by a half-time ($T_3$) of about 160 hours. Mean values of these half-disappearance times for both groups of patients, as derived from their respective whole blood and plasma curves, based on the data collected over a two week period, are listed in Table II. The figures reproduced here (Fig. 1 and 2) cover only the initial 24 hours following the administration of the labeled amino acid to illustrate more clearly the early phases.

Fig. 2.—Resolution of the whole blood $^{35}S$ concentration curve obtained from a patient with multiple myeloma into two exponential components. $T_1$ and $T_3$ are the respective initial and late half-disappearance times. The various half-disappearance times given in the figures and Table II are actually based on graphs covering the two week period of observation ; for illustrative purposes, only the early values are presented here.

Fig. 3.—Resolution of the whole blood $^{35}S$ concentration curve obtained from a patient with a malignant neoplasm other than multiple myeloma into three exponential components. $T_1$, $T_2$ and $T_3$ are the corresponding initial, intermediate and late half-disappearance times.

*$^{35}S$ urinary excretion.*—The $^{35}$S excretion in the urine is relatively slow and about the same for both groups of patients (Table I). The output of the first day ranges from about 5 per cent of the dose to a maximum of about 20 per cent of the dose. From the second day on, the $^{35}$S is eliminated in the urine exponentially the average doubling-time for cumulative excretion being about 7·2 days (Fig. 4) By the end of the first week, about 30 per cent of the dose has been excreted.

*$^{35}S$-cystine spaces of dilution.*—The zero-time radiosulfate whole blood spaces and their respective ratios to body surface and to weight are given in Table I. These results are similar for both the myeloma patients and those having other neoplasms. The average space for patients with myeloma is 7·39 $\pm$ 1·41 liters, corresponding to 13·2 $\pm$ 3·49 $\times$ $10^{-2}$ liter per kilogram of body weight. For the

FIG. 4.—The cumulative mean $^{35}$S urinary excretion for the first week following the administration of the tagged cystine.

other patients, an average space of 8·66 $\pm$ 2·67 liters or 14·7 $\pm$ 4·04 $\times$ $10^{-2}$ liter per kilogram of body weight was found. As was noted earlier, since the $^{35}$S concentration in the whole blood and plasma are almost identical, the respective spaces are likewise very similar, and, for brevity, only the whole blood radiosulfate spaces are tabulated.

### DISCUSSION

The intravenous administration of $^{35}$S-l-cystine provides an immediate supply of this readily detected labeled amino acid to all sites of protein synthesis. The $^{35}$S labeled cystine exchanges its labeled position with the other amino acids only slightly, if at all. The sulfur of $^{35}$S-cystine is not transferred to methionine. It is believed that through the use of this amino acid the uptake and loss of isotope by the protein are almost exclusively in the form of the administered amino acid (Goldsworthy and Volwiler, 1957). The resulting turnover curve is usually divided into the following phases (Armstrong, Bronsky and Hershman, 1955 ; Golds-

TABLE I.—$^{35}S$ *Urinary Excretion and Zero-time Whole Blood Spaces of Dilution*

| Sex | Age | Diagnosis | % Urinary excretion: 1st day, First 8 hrs | % Urinary excretion: 1st day, Next 16 hrs | % Urinary excretion: 2nd day | % Urinary excretion: 3rd day | Space (liter) | $\frac{\text{Space}}{\text{Body surface}}\left(\frac{\text{liter}}{\text{meter}^2}\right)$ | $\frac{\text{Space}}{\text{Wt.}}\left(\frac{\text{liter}}{\text{kg}}\times 100\right)$ |
|---|---|---|---|---|---|---|---|---|---|
| | | Group A | | | | | | | |
| ♀ | 62 | Multiple myeloma | 8·30 | 6·30 | 5·65 | 4·44 | 9·75 | 6·17 | 16·6 |
| ♀ | 60 | ,, | 5.64 | 8·50 | 7·93 | 2·96 | 6·50 | 3·65 | 9·05 |
| ♀ | 66 | ,, | 2·38 | 2·86 | 6·30 | 2·09 | 7·50 | 4·78 | 13·4 |
| ♀ | 62 | ,, | 6·44 | 6·76 | 5·18 | 3·88 | 7·60 | 6·23 | 17·6 |
| ♀ | 51 | ,, | 12·5 | 5·03 | 5·42 | 4·59 | 5·62 | 3·53 | 9·51 |
| | | Average | 7·05 | 5·89 | 6·10 | 3·59 | 7·39 | 4·87 | 13·2 |
| | | | ±3·33* | ±1·88 | ±0·99 | ±0·94 | ±1·41 | ±1·17 | ±3·49 |
| | | Group B | | | | | | | |
| ♂ | 57 | Hypernephroma | 3·15 | 4·50 | 4·64 | 4·21 | 7·50 | 4·57 | 13·1 |
| ♀ | 53 | Carcinoma breast | 6·96 | 10·3 | 5·83 | 3·28 | 10·2 | 5·63 | 14·1 |
| ♂ | 45 | Brain tumour | 0·00 | 12·7 | 7·09 | 5·57 | 6·50 | 3·87 | 10·9 |
| ♀ | 63 | Papilloma breast | 12·7 | 8·95 | 7·14 | 2·15 | 5·95 | 4·16 | 13·1 |
| ♀ | 75 | Bronchogenic carcinoma | 3·53 | 5·65 | 3·60 | 2·18 | 13·1 | 7·80 | 22·5 |
| | | Average | 5·27 | 8·41 | 5·66 | 3·48 | 8·66 | 5·21 | 14·7 |
| | | | ±4·32 | ±2·99 | ±1·38 | ±1·30 | ±2·67 | ±1·58 | ±4·04 |

* Standard deviation.

worthy and Volwiler, 1957) : (a) rapid incorporation of the amino acid radioactivity into the protein and rapid release of the newly synthesized protein into the circulation in the first 24 hours ; (b) the distribution and mixing period, lasting a few days ; (c) the exponential portion of the curve, presumed to reflect primarily the rate of metabolic degradation of the labeled protein ; and (d), if the experiment continues for sufficient time, a phase reflecting the re-incorporation of the isotope into the protein.

In comparing the fate of tagged cystine with that of radiosulfate, some significant differences are observed. While the spaces of dilution for these two substances are quite similar and in accord with those reported for radiosulfate for normal subjects as well as for others having malignancies (Siegel *et al.*, 1959 ; Ryan *et al.*, 1956 ; Walser, Seldin and Grollman, 1953), the handling and turnover of these compounds are quite different. Fig. 1 illustrates the $^{35}$S whole blood concentration as a function of time for those receiving labeled cystine. Following administration of labeled cystine the $^{35}$S blood concentration decreases slowly ; thus, by the second day, when the $^{35}$S concentration following radiosulfate, as previously reported by us, is less than 0·5 per cent dose/liter, the $^{35}$S concentration from cystine is about six times greater. This markedly slow fall in $^{35}$S blood levels is characterized by the greatly prolonged late ($T_3$) half-disappearance times following labeled cystine (Table II) as compared to those found for radiosulfate.

TABLE II.—*Initial, Intermediate, and Late Half-disappearance Times obtained from $^{35}$S Whole Blood and Plasma Concentration Curves.*

| | Average half-disappearance times (hours) | | |
|---|---|---|---|
| Patients | $T_1$ | $T_2$ | $T_3$ |
| Multiple myeloma . | 0·448 ± 0·157* | — † | 281 ± 77 |
| Other neoplasms . | 0·265 ± 0·135 | 1·41 ± 0·65 | 162 ± 121 |

* Standard deviation.

† $T_2$ was found in two of the five multiple myeloma patients, averaging 2·47 hours.

Thus, the average value $T_3$ for patients with myeloma given $^{35}$S-cystine is 281 hours as compared to 15·9 hours following tagged sulfate. A similar lengthening of this parameter is observed also for the patients with other neoplasms. There is a corresponding lower output of $^{35}$S in the urine from $^{35}$S-cystine as compared to that following administration of radiosulfate (Fig. 4). By the second day, when more than 60 per cent of the injected sulfate has been excreted, those given $^{35}$S-cystine have excreted only about 20 per cent of the dose. Furthermore, for those given labeled cystine, an equilibrium is attained after the second day, the cumulative urinary output growing exponentially, with an average doubling-time, as noted earlier, of about 173 hours, which is in good agreement with the observed late half-disappearance times (Table II). Thus, after the second day, the constant fraction of the remaining dose, which disappears daily from the blood, was found in the urine, suggesting that the $^{35}$S is then being degraded and directly excreted.

When $^{35}$S-cystine is administered to control subjects, the label is incorporated into the normal serum albumin and globulins. When $^{35}$S-cystine is given to patients with myeloma, the label also is incorporated into the normal albumin and globulins as well as the abnormal myeloma globulins. The present investigation did not involve study of homologous serum proteins, and the curves obtained represent summation effects for all the serum protein complexes. It is possible that if individual globulins were studied, greater differences might have been observed between the turnover of the $^{35}$S-cystine labeled normal globulins and the labeled myeloma globulins (Putnam, 1959).

There is a suggestive finding of a difference in the fate of $^{35}$S-cystine for myeloma patients as compared to those having other malignancies. The initial ($T_1$) and late ($T_3$) half-disappearance times are longer for those having myeloma (Table II). This suggests that the hyperglobulinemia of myeloma may result from a slower catabolism of myeloma globulin. In this connection, Putnam and Hardy (1955) observed a greater half-disappearance time for the turnover of beta myeloma globulin than that obtained for the normal beta globulins by London (1950), and stated that the hyperglobulinemia of myeloma might be due to accumulation of the myeloma globulin by virtue of its slow turnover rate.

### SUMMARY

$^{35}$S-l-cystine (specific activity 30 mc/g.) in doses of approximately 0·5 mc was administered intravenously to five patients with multiple myeloma and to five subjects with other neoplastic diseases. For both groups of patients, the $^{35}$S concentration of whole blood and plasma were similar. The $^{35}$S blood levels declined slowly ; at five days after administration of the dose they were approximately three-quarters of the initial 8 hour values. The mean zero-time spaces of dilution were $7{\cdot}39 \pm 1{\cdot}41$ liters for the patients with multiple myeloma and $8{\cdot}66 \pm 2{\cdot}67$ liters for those with other neoplasms. $^{35}$S urinary excretion was low, 13·6 to 30·9 per cent of the dose appearing in the urine within 72 hours. The turnover and excretion of $^{35}$S administered as cystine are very much slower than when given as radiosulfate. By graphical analysis, it was possible to resolve the blood concentration curves into three exponential components. Patients with multiple myeloma were found to have longer initial and late half-disappearance times when compared to the subjects with other neoplastic diseases.

The authors wish to acknowledge the excellent technical assistance rendered by Messrs. Joseph V. Marino, Jack Sokol, and David Weinstein. This investigation was supported in part by Grant No. C-3438 from the National Cancer Institute of the National Institutes of Health, U.S. Public Health Service.

### REFERENCES

Armstrong, S. H., Jr., Bronsky, D. and Hershman, J.—(1955) *J. Lab. clin. Med.*, **46**, 857.

Goldsworthy, P. D. and Volwiler, W.—(1957) *Ann. N.Y. Acad. Sci.*, **70**, 26.

London, I. M.—(1950) The Robert Gould Research Foundation, Inc., Cincinnati, Symposium on Nutrition, **2**, 72.

Putnam, F. W.—(1959) *New Engl. J. Med.*, **261**, 902.

*Idem* and Hardy, S.—(1955) *J. biol. Chem.*, **212**, 371.

Ryan, R. J., Pascal, L. R., Inoye, T. and Bernstein, L.—(1956) *J. clin. Invest.*, **35**, 1119.

Sachs, B. A., Cady, P. and Ross, G.—(1954) *Amer. J. Med.*, **17**, 662.

Siegel, E., Sachs, B. A. and Graig, F. A.—(1959) *Proc. Soc. exp. Biol., N.Y.*, **101**, 53.

Walser, M., Seldin, D. W. and Grollman, A.—(1953) *J. clin. Invest.*, **32**, 299.